# Protective effect of Astragaloside IV on chronic intermittent hypoxia-induced vascular endothelial dysfunction through the calpain-1/SIRT1/AMPK signaling pathway

**DOI:** 10.3389/fphar.2022.920977

**Published:** 2022-08-02

**Authors:** Fang Zhao, Yan Meng, Yue Wang, Siqi Fan, Yu Liu, Xiangfeng Zhang, Chenyang Ran, Hongxin Wang, Meili Lu

**Affiliations:** Key Laboratory of Cardiovascular and Cerebrovascular Drug Research of Liaoning Province, Jinzhou Medical University, Jinzhou, China

**Keywords:** Astragaloside IV, chronic intermittent hypoxia, vascular endothelial dysfunction, oxidative stress, calpain-1

## Abstract

Vascular endothelial dysfunction (VED) is linked with the pathogenesis of obstructive sleep apnea (OSA) comorbidities, such as cardiovascular disease. Astragaloside IV (As-IV) has exhibited significant improvement for endothelial dysfunction. Nonetheless, the protective mechanism is not clear. Therefore, the present study investigated the potential mechanism of As-IV on VED. Calpain-1 knockout and wild-type C57BL/6 mice exposed to chronic intermittent hypoxia (CIH) were established and treated with As-IV (40, 80 mg/kg) for 4 weeks. Human coronary artery endothelial cells (HCAECs) subjected to CIH exposure were pretreated with As-IV, MDL-28170 (calpain-1 inhibitor) and SRT1720 (SIRT1 activator) for 48 h *in vitro*. The endothelial function, inflammation, oxidative stress and mitochondrial function were measured to evaluate VED. Our data revealed that As-IV treatment ameliorated CIH-induced endothelial-dependent vasomotion and augmented nitric oxide (NO) production. As-IV administration suppressed the secretion of inflammation, oxidative stress and mitochondrial dysfunction. As-IV treatment reduced the expression of calpain-1 and restored the downregulated expression of SIRT1 and Thr^172^ AMPK and Ser^1177^ eNOS phosphorylation. The effects of calpain-1 knockout and SRT1720 were similar to the effect of As-IV on VED. These findings demonstrated that As-IV ameliorated VED induced by chronic intermittent hypoxia via the calpain-1/SIRT1/AMPK signaling pathway.

## Introduction

Obstructive sleep apnea (OSA) is a sleep breathing disorder that is manifested as the partial or complete collapse of the upper airway during sleep (Baltzis et al., 2016). OSA affects 14% and 5% of adult males and females in the US, respectively (Chang et al., 2020), and it is an independent and significant factor of cerebrovascular and cardiovascular disease (CVD). Previous studies showed that chronic intermittent hypoxia (CIH) was the major pathophysiological characteristic of vascular endothelial dysfunction (VED) in OSA, which may lead to CVD by initiating nitric oxide (NO) unavailability, oxidative stress and vascular inflammation (Feng et al., 2012). As the first-line treatment of OSA patients, continuous positive airway pressure (CPAP) has a low compliance rate and poor prevention effect on cardiovascular events (Mcevoy et al., 2016; Arnaud et al., 2020). Therefore, there is an urgent need for other treatments to acquire an improvement in preventing cardiovascular complications and quality of life of OSA patients.

SIRT1 is an NAD^+^-dependent deacetylase that has crucial role in mediating inflammation, oxidative stress and mitochondrial function (Mihanfar et al., 2021). SIRT1 is involved in maintaining vascular endothelial homeostasis and protecting VED (Ministrini et al., 2021). SIRT1 and AMPK stimulate NO production by increasing endothelial NO synthase (eNOS) activity (Xia et al., 2014). Calpain is a conserved family of calcium-activated cysteine proteases that widely exist in mammals. Calpain-1, is the major isoform, and it is primarily expressed in endothelial cells and participates in many pathophysiological processes (Liu et al., 2020). Early studies showed that VED was associated with calpain overactivation in chronic vascular diseases (Miyazaki and Miyazaki, 2018). Calpain-1 also contributes to oxidative stress, inflammation and adhesiveness to leukocytes (Xu C. et al., 2021). Calpain is involved in the AMPK/eNOS signaling pathway in VED (Etwebi et al., 2018). Although previous studies demonstrated that activation of calpains caused a reduction in SIRT1 protein levels (Biel et al., 2016), whether the calpain-1/SIRT1/AMPK signaling pathway is a major factor of VED was not reported.

Astragaloside IV (As-IV) is a major natural molecule compound isolated from *Astragalus* membranaceus that exerts diverse cardiovascular pharmacological activities, including anti-inflammatory, anti-fibrosis, anti-oxidative stress and immune regulation activities (Chen et al., 2021; Deng et al., 2022). Our previous studies demonstrated that As-IV protected against hyperglycemia-induced VED by inhibiting calpain-1 overactivation (Nie et al., 2019). As-IV also attenuates CIH-induced myocardial injury and inflammation (Chen et al., 2020; Jiang et al., 2020). However, the potential mechanism of As-IV activity against CIH-induced VED is not clear. Therefore, we used calpain-1 knockout *in vivo* and *in vitro* to further study the effect of As-IV on CIH-induced VED and the potential mechanism.

## Materials and methods

### Reagents

As-IV were obtained from Nanjing Jingzhu Biotechnology Co. Ltd. (Nanjing, China). Assay kits for Cell Counting Kit-8, NO nitrate reductase, dihydroethidium (DHE), GSH-px, MDA, SOD, JC-1, DAF-FM diacetate (DAF-FM DA) and MitoSOX were obtained from Beyotime Biotechnology (Shanghai, China). Calpain-1, SIRT1 and *β*-actin were obtained from Proteintech (Wuhan, China). Ser^1177^ eNOS phosphorylation, total endothelial nitric oxide (eNOS), Thr^172^ AMPK phosphorylation, total 5’ AMP activated protein kinase (t-AMPK), vascular cell adhesion molecule 1 (VCAM-1) and intracellular adhesion molecule 1 (ICAM-1) were obtained from ABclonal (Wuhan, China). Phenylephrine (PE) and acetylcholine (Ach) were obtained from Sigma Aldrich (Shanghai, China). MDL-28170, SRT1720 and L-NAME were obtained from Selleck (Houston, TX, United States).

### Animal experiments

Eight-to twelve-week-old male wild-type mice (Liaoning Changsheng Biotechnology Co. Ltd.) and mice deficient in calpain-1 (Capn1 EK684^−/−^, Cyagen Biosciences Inc.) with a mean body weight of 25 g were used. Both wild-type and Capn1^−/−^ mice are the C57BL/6 background, and the same strain. All mice were grown in a normal environment at a controlled temperature (25 ± 2°C) under a 12-h light/dark cycle. The Animal Ethics Committee of Jinzhou Medical University approved all animal procedures. Wild-type mice were randomly divided into four groups (*n* = 10): control group (Con), chronic intermittent hypoxia group (CIH), CIH + As-IV 40 mg/kg/d (AL) and CIH + As-IV 80 mg/kg/d (AH). Capn1^−/−^ mice were also divided randomly into two groups (*n* = 10): the control group (CK) and the chronic intermittent hypoxia group (MK). The model of CIH exposure established in this study was reported previously (Jiang et al., 2020). Briefly, the mice were exposed to cages of automated, computer-controlled O_2_ concentration exchange systems (Proox-100, Shanghai Tow-Int Tech Ltd.) to achieve a similar oxyhemoglobin saturation (SpO_2_) nadir (50∼60%) in moderate to severe patients based on previous studies (Zhou et al., 2014; Hu et al., 2021; Li et al., 2021). The mice were exposed to 20 hypoxic events/h (90 s of 21% O_2_ and 90 s of 10% O_2_) for 8 h/day for 4 weeks. The control mice breathing normal gas were housed in the same environment. During the CIH interval, mice received a gastric injection of As-IV (dissolved in 5% sodium carboxymethyl cellulose) at 40 or 80 mg/kg per day for 4 weeks. The special gavage needle for mice is used for intragastric administration of mice, and the volume of intragastric administration does not exceed 0.2 ml. After 4 weeks, corresponding samples (thoracic aortas and serum) were collected for subsequent experiments.

### Cell culture

Human coronary artery endothelial cells (HCAECs) were purchased from BLUEFBIO and grown in DMEM supplemented with 15% fetal bovine serum (HyClone, Logan, Utah, United States) in a CO_2_ incubator as described previously (Lv et al., 2019). The model of CIH in HCAECs was implemented by alternating cycles of 1% O_2_ for 5 min followed by 20% O_2_ for 5 min (3 cycles/h) at 37°C for 24 h in a chamber through automated, computer-controlled O_2_ concentration exchange systems (GC-CT, Heilongjiang MAWORDE Industry and Trade Ltd.) as described previously (Jiang et al., 2020; Xu H. et al., 2021; Yan et al., 2021). Cells were incubated with As-IV (100 µM), MDL-28170 (calpain-1 inhibitor, 20 µM) and SRT1720 (SIRT1 activator, 4 µM) for 48 h before CIH exposure.

### Vascular reactivity

After sacrifice, the thoracic aorta was fully exposed, quickly removed and placed in ice-cold physiological salt solution (PSS) that included the following: NaCl (130 mM), KCl (4.7 mM), KH_2_PO_4_ (1.18 mM), MgSO_4_ (1.17 mM), CaCl_2_ (1.16 mM), NaHCO_3_ (14.9 mM), EDTA (0.026 mM), and glucose (11.1 mM). The thoracic aorta was cut into rings approximately 2 mm in length without fat and connective tissue under a microscope. Changes in the tension of rings were measured using DMT (720 M, Danish Myotechnology, AÅrhus, Denmark). The rings of all groups were incubated in chambers with 95% O_2_ and 5% CO_2_ at 37 °C. Before the formal experiment, we used K-PSS (60 mM KCl in PSS solution; equimolar substitution of KCl for NaCl) to activate the rings. If the difference change in tension of rings was less than 10%, the vasoconstriction was considered relatively stable and rings were used in subsequent experiments. The rings were precontracted with PE (10^−5^ M), and ACh was added after the contraction was stable in the range of concentration (10^−8^ M - 10^−5^ M). The endothelium-dependent relaxation (EDR) response to ACh is expressed as the percentage of maximal contraction amplitude induced by PE. To test the effect of NO on vascular relaxation, the aortic rings of all groups were incubated with the nitric oxide synthase inhibitor NG-nitro-l-arginine (L-NAME, 100 µM) for 30 min before PE-induced constriction. To verify the role of SIRT1 on VED, the rings from the control group and the CIH group were incubated with SRT1720 (4 μM, dissolved in DMSO) for 24 h in chambers with 95% O_2_ and 5% CO_2_ at 37°C. DMSO (0.1% v/v) did not modify agonist-induced responses. Following incubation, the rings were used to measure vascular reactivity as mentioned above.

### Determination of intracellular ROS production and NO imaging

The level of intracellular ROS in aortas and HCAECs was detected using DHE staining. Frozen sections of aortas (embedded in OCT and 5 μm thick) and cells were incubated with 5 µM DHE for 30 min at room temperature in the dark. Rosup was used as a positive control to detect intracellular ROS expression. The sections and cells were stained with Hoechst 33342 for 4 min and washed 3 times with PBS. NO imaging experiments in aortas and cells were performed as described previously (Lee et al., 2020). Briefly, frozen sections of aortas and cells were incubated with 20 µM DAF-FM DA for 20 min at 37°C. The sections were stained with Hoechst 33342 for 4 min. Images were viewed under a Leica DMI3000B fluorescence microscope, and the relative fluorescence intensity was analyzed using ImageJ software.

### Immunofluorescence staining

Four% paraformaldehyde-fixed, paraffin-embedded aortic tissues were cut into 5-μm thick slices. Then the slices were deparaffinized, rehydrated, and incubated in 5% BSA for 1 h. The slices were incubated with primary antibodies against calpain-1 (1:100) and SIRT1 (1:100) at 4°C overnight. The relative secondary antibody was incubated with the slices for 1 h at 37°C in the dark. Nuclei were marked using Hoechst 33342. Quantification of the images was analyzed by ImageJ Software from each group.

### Western blot

The collected sample proteins were homogenized in lysis buffer, and the concentration of total proteins was measured using the BCA method. The prepared samples were separated using 8%–10% SDS–PAGE then transferred to PVDF membranes. Membranes were blocked with 1% BSA for 1.5 h and subsequently incubated with calpain-1 (1:1,000), SIRT1 (1:4,000), t-AMPK (1:1,000), Thr^172^ phosphorylated AMPK (1:1,000), eNOS (1:1,000), ICAM-1 (1:1,000), VCAM-1 (1:1,000), Ser^1177^ phosphorylated eNOS (1:1,000) and *β*-actin (1:10,000) overnight at 4°C. After washing with TBST, membranes were incubated with HRP-conjugated secondary antibody for 2 h at room temperature. The intensity of the bands was determined using ImageJ software.

### Measurements of SOD, MDA, GSH-px

MDA, SOD, and GSH-px assay kits were used to measure MDA and GSH-px levels and SOD activity in the serum and culture supernatant according to the manufacturer’s protocol. The NO levels in serum and culture supernatant were tested using the nitrate reductase method according to the manufacturer’s protocol.

### Enzyme-linked immunosorbent assay

The levels of IL-6 and TNF-α in serum and culture supernatant were analyzed using ELISA kits according to the manufacturer’s instructions.

### Cell viability assay

The cell viability of HCAECs was assessed using a Cell Counting Kit-8 (CCK-8 kit) as previously described (Yan et al., 2021). Briefly, HCAECs under the indicated times and different conditions were seeded in 96-well plates at the density of 2×10^4^ cells/well and were added with 10 µl CCK-8 reagent for 1 h. The absorbance at 450 nm was measured with a microplate reader (BioRad, United States). Cell viability was calculated based on the percentage of the optical density relative to that of untreated controls.

### Assessment of mitochondrial function

The cells were incubated with 10 μg/ml JC-1 and 5 µM MitoSOX. The mitochondrial membrane potential was determined using JC-1 staining as previously described (Umbaugh et al., 2021). Briefly, the cells were incubated with JC-1 at 10 μg/ml for 15 min at 37°C, CCCP was set as the positive control of mitochondrial membrane potential decline, and then the images were observed by fluorescence microscopy. Increased monomer (green) fluorescence levels and decreased J-aggregate (red) fluorescence levels indicate mitochondrial membrane potential collapse. All images were analyzed by ImageJ software. Mitochondrial reactive oxygen species (mitoROS) were determined using MitoSOX as previously described (Zhou et al., 2021). Briefly, the cells were incubated with 5 µM MitoSOX for 10 min at 37°C in the dark. Then the images were captured using fluorescence microscopy and analyzed using ImageJ software.

### Statistical analysis

All the data are represented as the means ± standard deviation (SD) and analyzed by SPSS 26.0 software. Statistical analyses of all data were performed using One-way analysis of variance (ANOVA) followed by Duncan’s LSD. *p* < 0.05 was considered statistically significant.

## Results

### Effects of As-IV, Capn1 knockout and SRT1720 on VED in mice exposed to CIH

**FIGURE 1:**
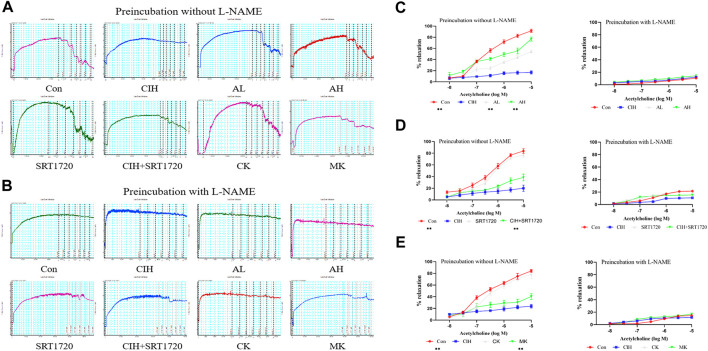
Effects of As-IV, Capn1 knockout and SRT1720 on VED in mice exposed to CIH. **(A,B)** The reactivity of PE-preconstricted aortic rings from all groups to ACh-mediated relaxation with or without L-NAME **(C)** The effect of As-IV treatment on ACh-induced vasodilation in aortas of CIH-exposed (90 s of 21% O_2_ and 90 s of 10% O_2_) mice (*n* = 5). **(D)** The effect of SRT1720 (4 µM) incubation on ACh-induced vasodilation in aortas of CIH-exposed mice (*n* = 5). **(E)** The effect of Capn1 knockout treatment on ACh-induced vasodilation in aortas of CIH-exposed mice (*n* = 5). Data are presented as the means ± SD, **p < 0.01, vs. CIH group.

EDR is the most widely used assay for the assessment of VED (Figures 1A, B). The reactivity of isolated thoracic aortic rings to ACh was evaluated in CIH-exposed mice after treatment with As-IV for 4 weeks. As shown in Figure 1C, As-IV attenuated the damaged ACh-induced EDR in isolated thoracic aortic rings of mice exposed to CIH. To determine the effect of SIRT1 on CIH-induced VED, aortic rings from the control group and CIH group were incubated with SRT1720 (4 µM) for 24 h *in vitro*. Our studies showed that SRT1720 ameliorated relaxation to ACh in CIH-exposed mice (Figure 1D). To investigate the role of calpain-1 on CIH-induced VED, we detected ACh-induced dilation after PE preconstriction in the CK group and MK group. Our data indicated that the aortic rings from Capn1^−/−^ mice slightly but statistically improved CIH-induced VED compared to CIH-exposed mice (Figure 1E). And vascular relaxation to ACh was completely abolished by eNOS inhibitor L-NAME in all groups.

### Effects of As-IV and Capn1 knockout on CIH-induced oxidative stress and NO levels

**FIGURE 2:**
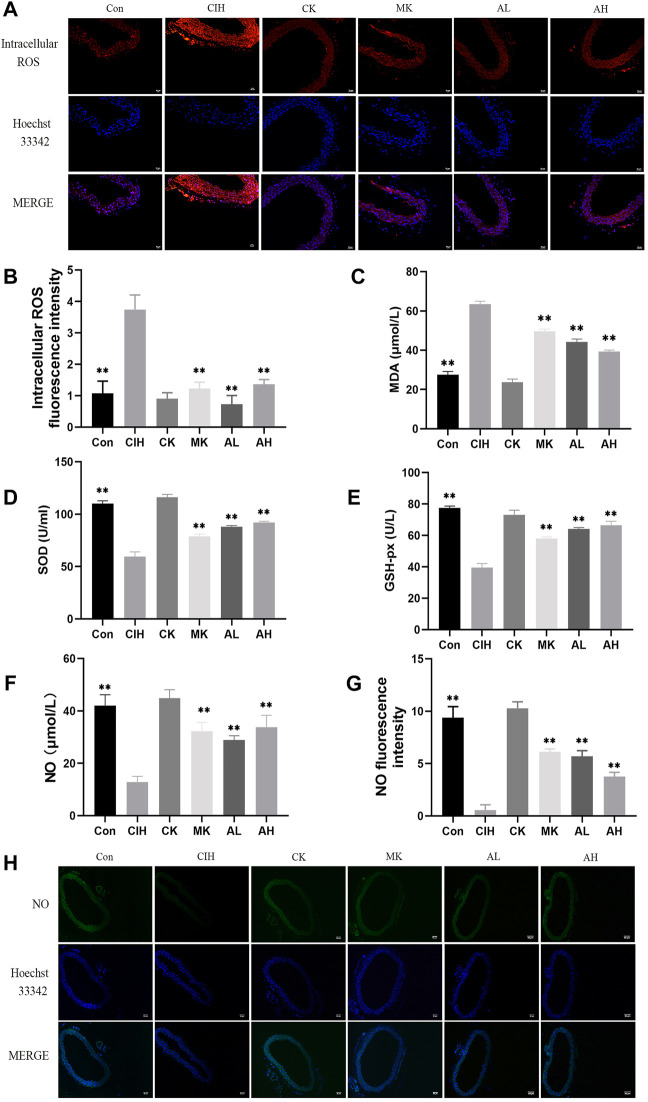
Effects of As-IV and Capn1 knockout on CIH-induced oxidative stress and NO levels. **(A,B)** The level of intracellular ROS in aortas was measured using DHE staining (×200 magnification; *n* = 3). DHE dye (red), Hoechst 33342 (blue). **(C–E)** The activity of SOD and the levels of MDA and GSH-px in serum were determined using kits (*n* = 8). **(F)** NO production in serum was evaluated using the nitrate reductase method (*n* = 8). **(G,H)** The level of NO in aortas was measured using DAF-FM DA fluorescence staining (×100 magnification; *n* = 3). DAF-FM DA dye (green), Hoechst 33342 (blue). Data are presented as means ± SD. ns. **p < 0.01, vs. CIH group.

To verify the effect of As-IV on VED in mice exposed to CIH, DHE staining, MDA, SOD, and GSH-px assay kits and the nitrate reductase method were performed. Compared to the control group, the levels of intracellular ROS and MDA were increased, the activity of SOD and the levels of GSH-px and NO were significantly inhibited in the CIH group. After treatment with As-IV (40 or 80 mg/kg) for 4 weeks, the activity of SOD and the production of NO and GSH-px were higher than the CIH group. The levels of intracellular ROS and MDA were lower than the CIH group. The effect of Capn1 knockout mice was similar to the above effect of As-IV (Figure 2).

### Effects of As-IV and Capn1 knockout on CIH-induced inflammation

**FIGURE 3:**
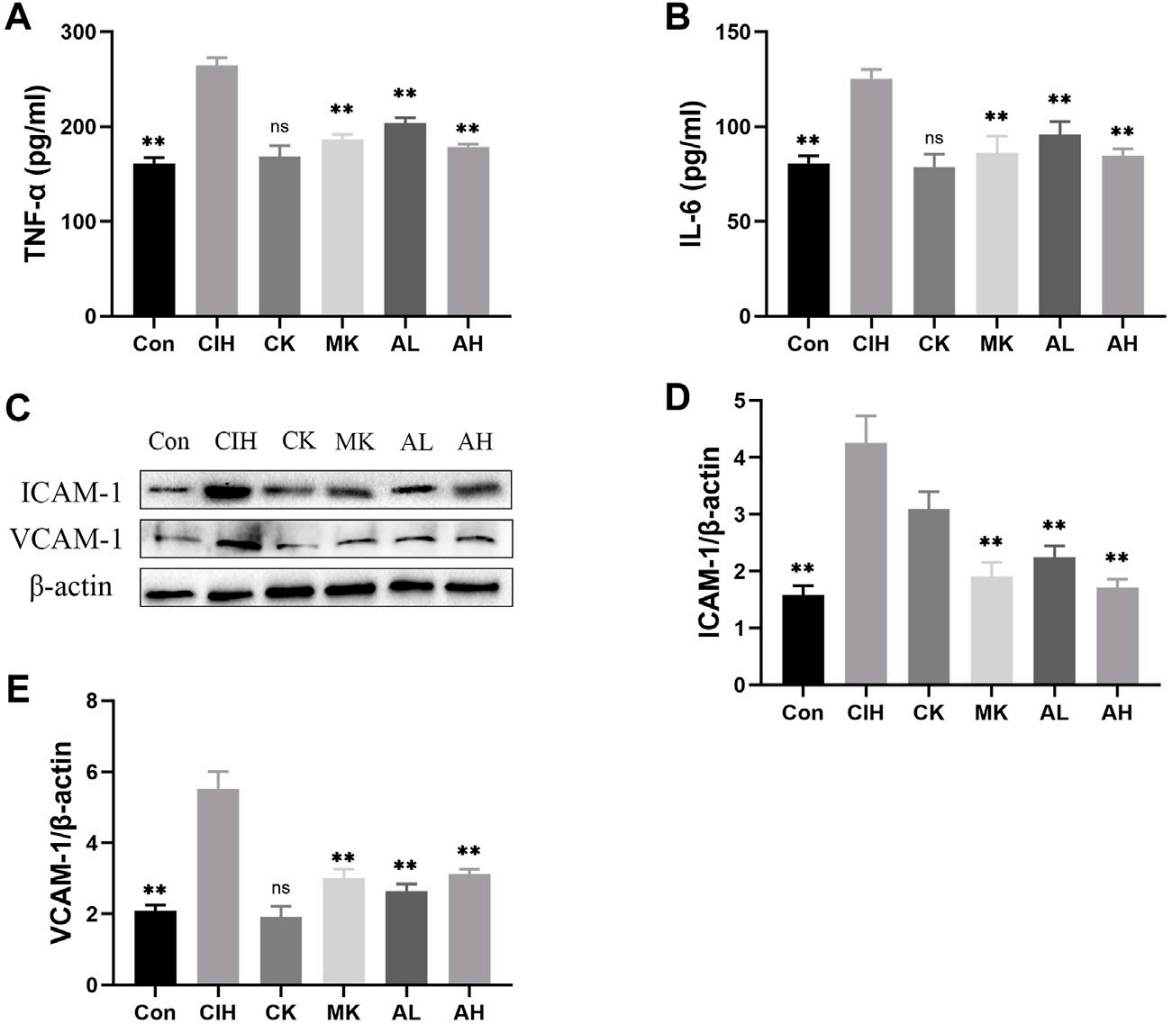
Effects of As-IV and Capn1 knockout on CIH-induced inflammation. **(A,B)** The levels of TNF-α and IL-6 were detected using ELISA (*n* = 8). **(C–E)** Quantitative Western blot analysis of the protein levels of VCAM-1 and ICAM-1 (*n* = 3). Data are presented as means ± SD. **p < 0.01, vs. CIH group.

Inflammation and adhesion molecules, such as VCAM-1 and ICAM-1, are vital for the development of VED. Our data indicated that the contents of TNF-α and IL-6 were enhanced in the CIH group, and As-IV and Capn1 knockout inhibited the levels of inflammation (Figures 3A, B). The mice exposed to CIH showed high expression of VCAM-1 and ICAM-1, and As-IV treatment and Capn1 knockout significantly abolished these changes at the protein level (Figures 3C-E).

### Effects of As-IV and Capn1 knockout on calpain-1, SIRT1, AMPK, and eNOS protein expression in CIH-induced mice

**FIGURE 4:**
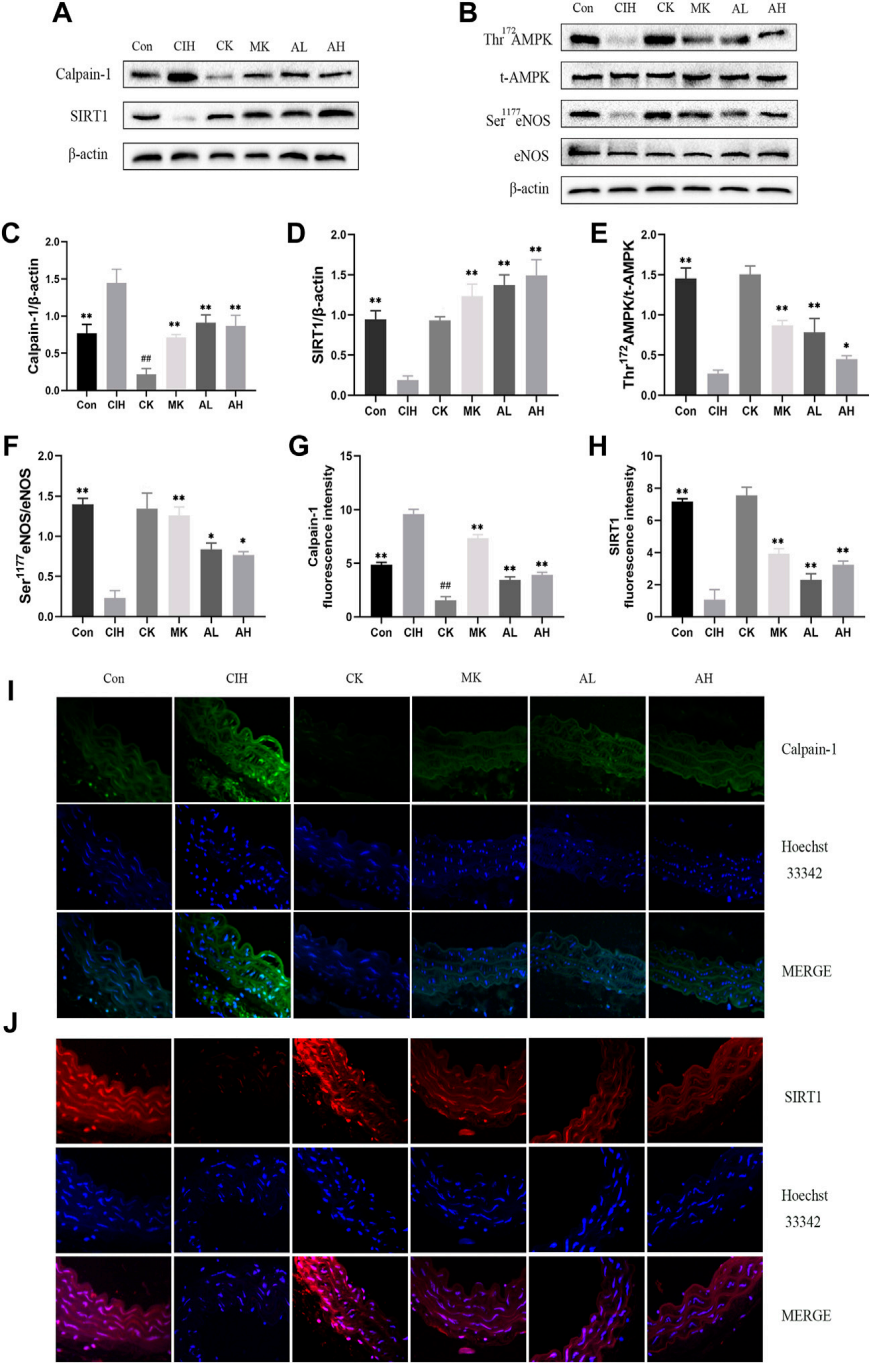
Effects of As-IV and Capn1 knockout on the SIRT1/AMPK/eNOS signaling pathway in CIH-induced mice. **(A–F)** Western blot analysis of calpain-1, SIRT1, Thr^172^ AMPK phosphorylation, t-AMPK, Ser^1177^ eNOS phosphorylation and eNOS protein expression in all groups with quantification (*n* = 3). **(G–J)** Representative fluorescence images and fluorescence intensity of calpain-1 and SIRT1 in all groups of aortic sections (×400 magnification; *n* = 3). Calpain-1 (green), SIRT1 (red), Hoechst 33342 (blue). Data are presented as means ± SD. #P < 0.05, vs. Con group, **p < 0.01, *P < 0.05, vs. CIH group.

To investigate whether calpain-1 regulates VED via the SIRT1/AMPK/eNOS signaling pathway in mice exposed to CIH, western blot analysis and immunofluorescence staining were conducted. Our findings revealed that compared with the control group, the expression level of calpain-1 was only reduced in the CK group, the other indexes were not statistically significant. CIH resulted in higher levels of calpain-1 and lower levels of SIRT1, Thr^172^ AMPK phosphorylation and Ser^1177^ eNOS phosphorylation. In contrast, treatment with As-IV and Capn1 knockout downregulated the level of calpain-1 and upregulated the levels of SIRT1, Thr^172^ AMPK phosphorylation and Ser^1177^ eNOS phosphorylation (Figure 4).

### Effects of As-IV, MDL-28170 and SRT1720 on VED in HCAECs subjected to CIH

**FIGURE 5:**
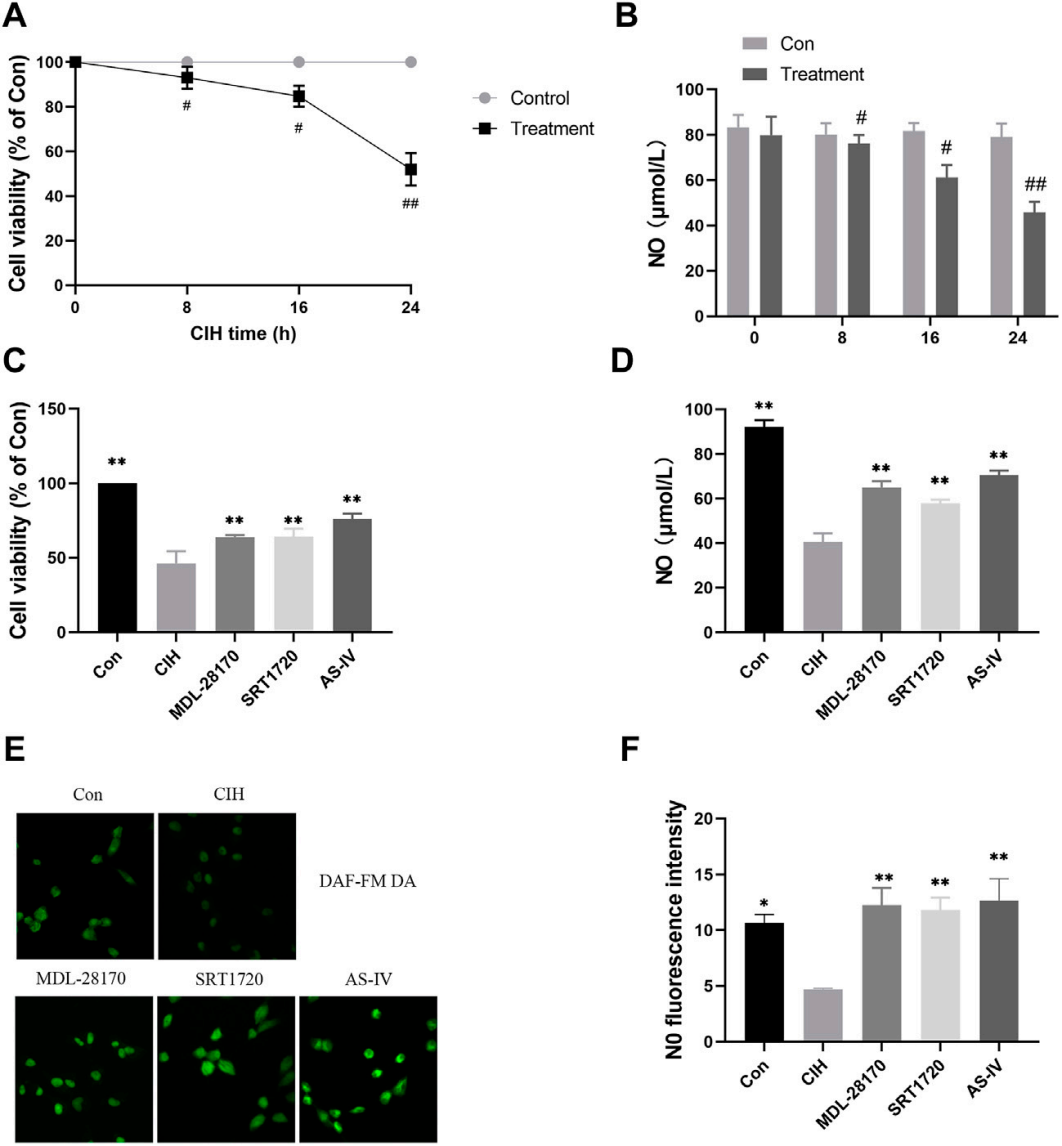
Effects of As-IV, MDL-28170 and SRT1720 on HCAEC viability and the production of NO. **(A)** Cell viability of HCAECs subjected to CIH (1% O_2_ for 5 min–20% O_2_ for 5 min) at the indicated times at the indicated times (*n* = 5). **(B)** NO level in HCAEC supernatant subjected to CIH at the indicated times (*n* = 8). **(C)** Cell viability of HCAECs in different groups (*n* = 5). **(D)** NO levels in HCAEC supernatants from different groups (n = 8). **(E,F)** Representative images and fluorescence intensity of NO in HCAECs of different groups (×200 magnification; n = 3). DAF-FM DA dye (green). Data are presented as means ± SD. ^##^ *p* < 0.01, ^#^P < 0.05, vs. Con group, **p < 0.01, *P < 0.05, vs. CIH group.

To test the effect of As-IV on VED, we measured the viability of HCAECs under normoxia or CIH exposure for the indicated times. Compared to the control group, cell viability decreased with prolonged CIH administration (Figure 5A). The NO level in the HCAEC supernatant after CIH exposure was also reduced in a time-dependent manner (Figure 5B). However, the As-IV, MDL-28170 and SRT1720 groups exhibited increased cell viability and NO levels in HCAECs compared to the CIH group (Figures 5C–F).

### Effects of As-IV, MDL-28170 and SRT1720 on oxidative stress in HCAECs exposed to CIH

**FIGURE 6:**
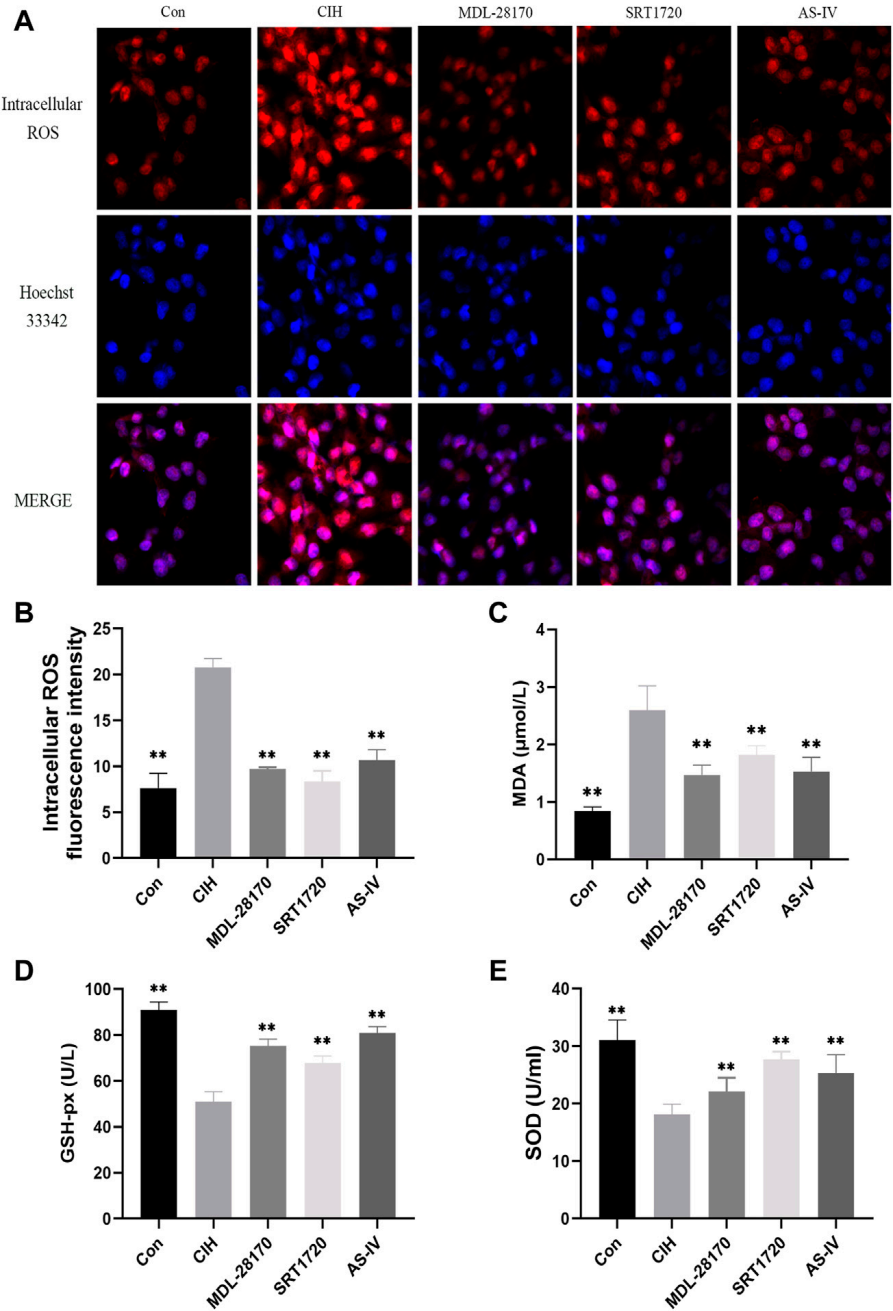
Effects of As-IV, MDL-28170 and SRT1720 on oxidative stress in HCAECs exposed to CIH. **(A,B)** Representative fluorescence images and fluorescence intensity of intracellular ROS in HCAECs of different groups (×200 magnification; *n* = 3). DHE dye (red), Hoechst 33342 (blue). **(C–E)** The activity of SOD and the levels of MDA and GSH-px in HCAEC supernatant were detected using kits (*n* = 8). Data are presented as means ± SD. **p < 0.01, vs. CIH group.

Oxidative stress is an indispensable element of the earlier periods of endothelial damage. Our data indicated that compared to the control group, CIH exposure facilitated the levels of intracellular ROS and MDA in HCAECs and decreased the level of GSH-px and the activity of SOD. The administration of AS-IV suppressed the levels of intracellular ROS and MDA in HCAECs compared to the CIH group and enhanced the activity of SOD and the level of GSH-px. The effect of AS-IV on oxidative stress was similar to MDL-28170 and SRT1720 (Figure 6).

### Effects of As-IV, MDL-28170 and SRT1720 on mitochondrial dysfunction in HCAECs exposed to CIH

**FIGURE 7:**
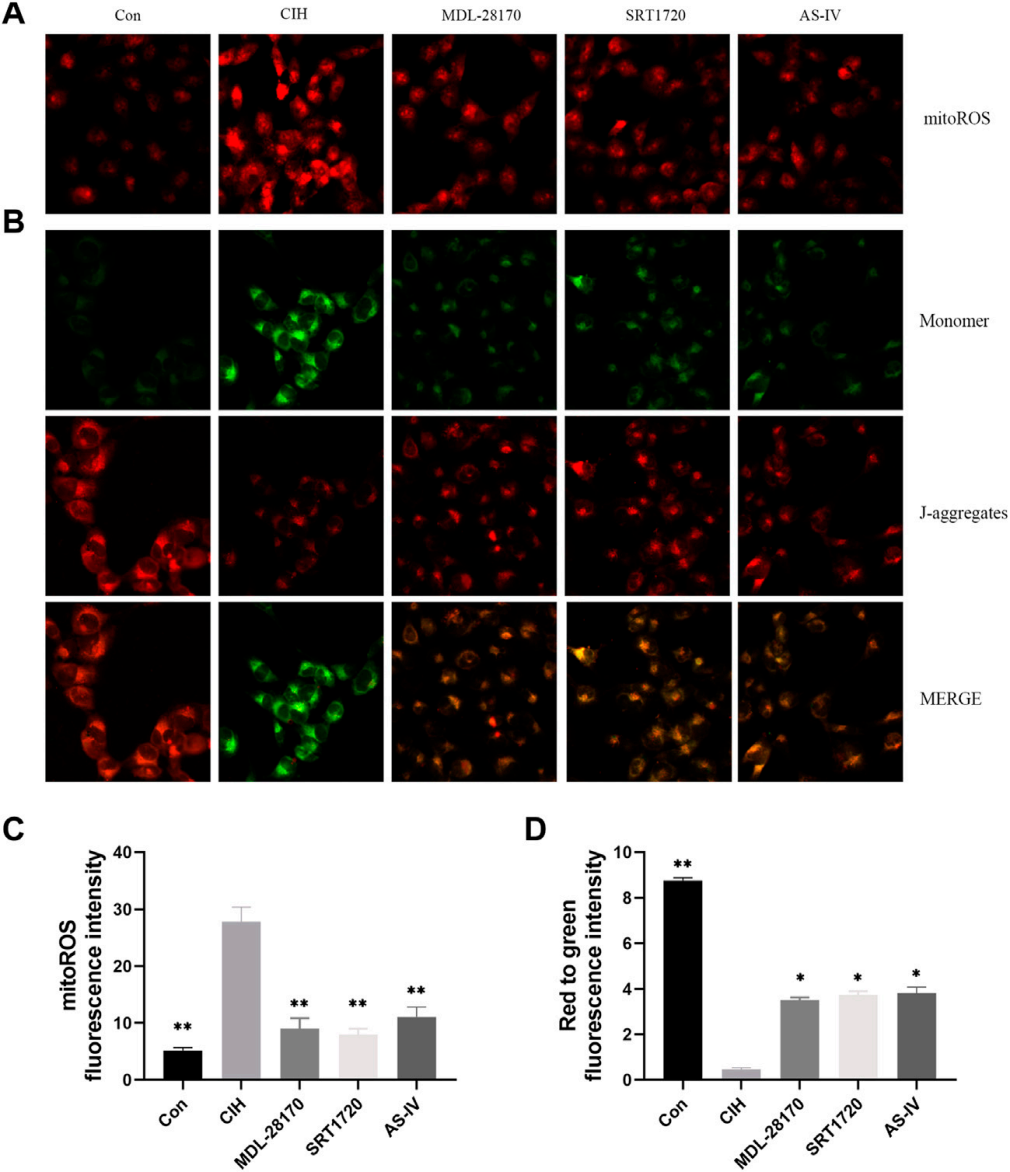
Effects of As-IV, MDL-28170 and SRT1720 on mitochondrial dysfunction in HCAECs exposed to CIH. **(A,C)** Representative fluorescence images and fluorescence intensity of mitoROS in HCAECs of different groups (×200 magnification; *n* = 3). MitoSOX dye (red). **(B,D)** Representative fluorescence images of JC-1 staining indicating the mitochondrial membrane potential in HCAECs of different groups (×200 magnification; *n* = 3). Monomer (green), J-aggregates (red). Data are presented as means ± SD. **p < 0.01, *P < 0.05, vs. CIH group.

Mitochondria are the primary producers of intracellular ROS. To test whether As-IV ameliorated CIH-induced mitochondrial dysfunction, JC-1 and MitoSOX staining were conducted. The results showed an apparent increase in the level of mitoROS and a decline in the membrane potential of HCAECs exposed to CIH compared to the control group. As-IV, MDL-28170 and SRT1720 treatment restored the increased level of mitoROS and the reduced membrane potential (Figure 7).

### Effects of As-IV, MDL-28170 and SRT1720 on inflammation in HCAECs exposed to CIH

**FIGURE 8:**
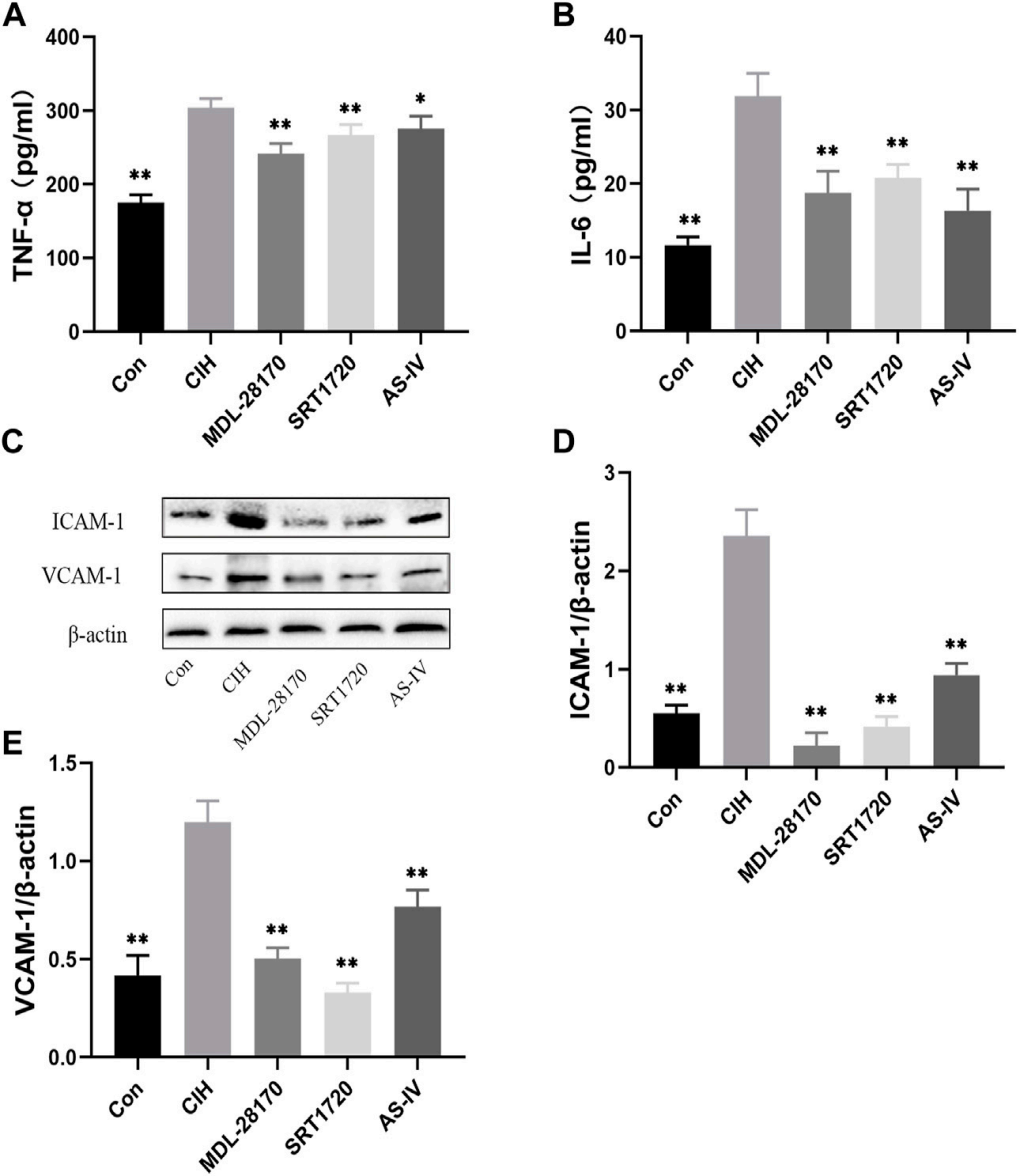
Effects of As-IV, MDL-28170 and SRT1720 on inflammation in HCAECs. **(A,B)** The levels of TNF-α and IL-6 was detected using ELISA (*n* = 8). **(C–E)** The levels of VCAM-1 and ICAM-1 were measured using Western blot (*n* = 3). Data are presented as means ± SD. **p < 0.01, vs. CIH group.

To evaluate the effect of As-IV, MDL-28170 and SRT1720 on inflammation, ELISA and Western blot experiments were performed. ELISA indicated that the levels of TNF-α and IL-6 was increased in the supernatants of HCAECs subjected to CIH, and As-IV, MDL-28170 and SRT1720 treatment reduced the secretion of inflammatory factors (Figures 8A, B). The levels of VCAM-1 and ICAM-1 protein were enhanced by CIH exposure compared to the control, and the administration of As-IV, MDL-28170 and SRT1720 partially abolished the upregulated secretion of inflammatory factors in HCAECs exposed to CIH (Figures 8C–E).

### Effects of As-IV, MDL-28170 and SRT1720 administration on calpain-1, SIRT1, AMPK, and eNOS protein expression in HCAECs

**FIGURE 9:**
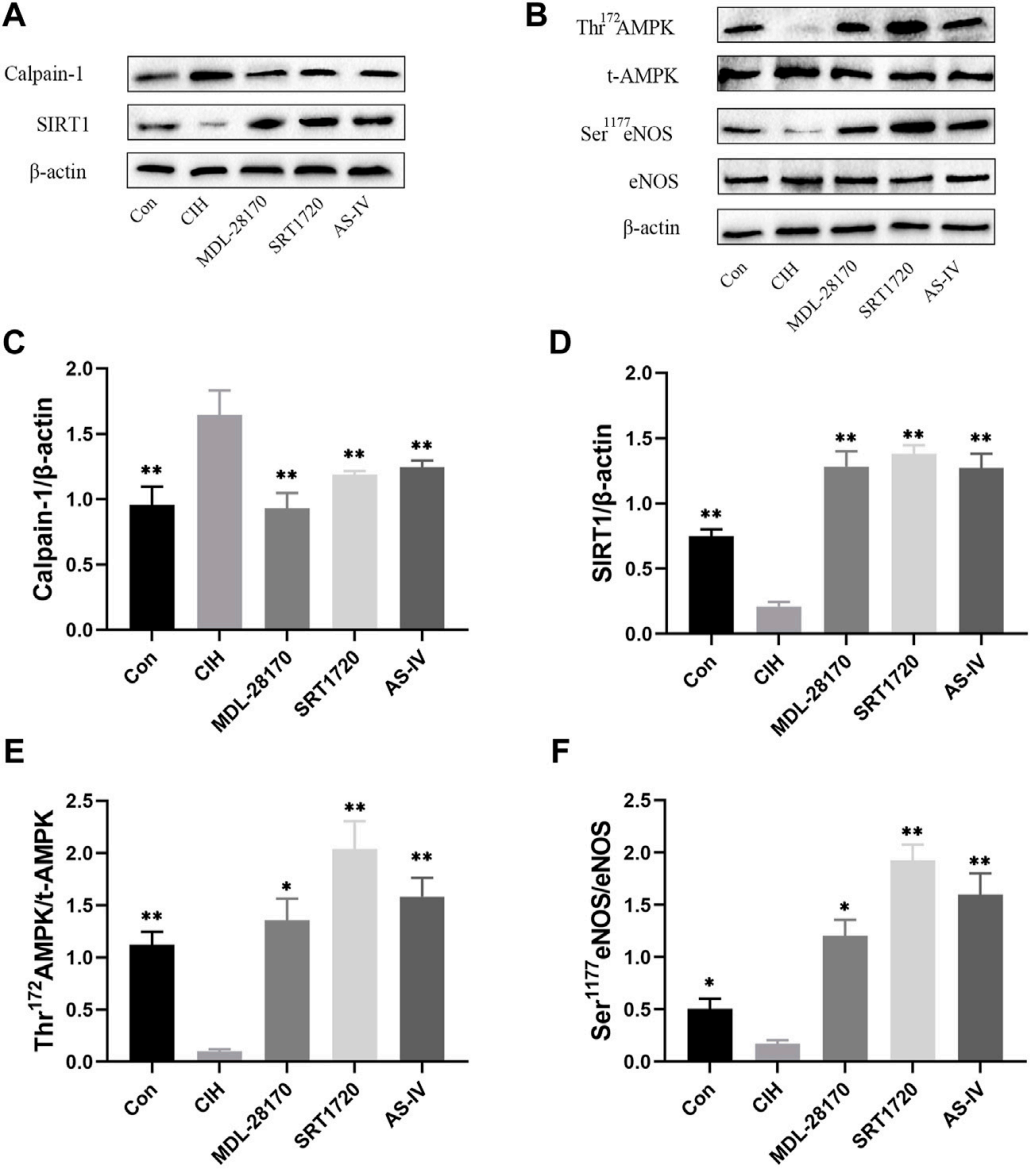
Effects of As-IV, MDL-28170 and SRT1720 administration on calpain-1, SIRT1, AMPK, and eNOS protein expression in HCAECs. **(A–F)** The protein expression levels of calpain-1, SIRT1, Thr^172^ AMPK phosphorylation, t-AMPK, Ser^1177^ eNOS phosphorylation and eNOS were analyzed using Western blots (*n* = 3). Data are presented as means ± SD. **p < 0.01, *P < 0.05, vs. CIH group.

To verify the effects of As-IV, MDL-28170 and SRT1720 administration on calpain-1, SIRT1, AMPK, and eNOS protein expression in HCAECs, Western blot experiments were performed. The data indicated that the expression level of calpain-1 protein was increased and the levels of SIRT1 protein expression, Thr^172^ AMPK phosphorylation and Ser^1177^ eNOS phosphorylation were reduced in HCAECs after CIH exposure. Treatment with As-IV, MDL-28170 and SRT1720 reversed the protein expression levels (Figure 9).

## Discussion

The vascular endothelium is formed from a monolayer of endothelial cells that regulate vascular hemostasis, maintains permeability and controls vascular tone. VED is linked with reduced nitric oxide (NO) production or bioavailability and damaged endothelial-dependent vasomotion (Cyr et al., 2020; Xu S. et al., 2021). OSA characterized by intermittent hypoxemia, is a main independent and important risk factor for the occurrence of cardiovascular disease (CVD). Numerous studies demonstrated that VED played a key role in the pathogenesis of OSA comorbidities, such as cardiovascular and metabolic diseases (Baltzis et al., 2016). After 4 weeks of exposure to intermittent hypoxemia in mice, we found that CIH decreased endothelial-dependent vasomotion to ACh, and As-IV reversed this response. CIH inhibited relative viability, Ser^1177^ eNOS phosphorylation and NO production in HCAECs, and As-IV rescued these changes. HCAECs were used in the current study since coronary endothelium expresses a pathologic gene pattern compared to aortic endothelium, and were commonly used in cell model of CIH (Dancu and Tarbell, 2007; Zychowski et al., 2016). However, this is also the limitation of this study. The differences between different species and tissues should be taken into account in future studies. These results suggested that As-IV ameliorated CIH-induced VED via the NO pathway. OSA is associated with the excessive generation of inflammatory mediators and the overproduction of adhesion molecules in the development of VED (Baltzis et al., 2016). Previous studies indicated that increased levels of the inflammatory factors TNF-α and IL-6 are primarily responsible for inflammatory responses in OSA patients (Kiernan et al., 2016). The upregulation of adhesion molecules, such as ICAM-1 and VCAM-1, stimulates endothelial activation by promoting leukocyte adhesion to endothelial cells when inflammation occurs (Sun et al., 2019). An activated endothelium results in decreased NO bioavailability and impaired vascular tone, which further cause VED. Intermittent hypoxia accelerated the activation of ICAM-1 and VCAM-1, which was demonstrated in human endothelial cells and animal models (Feng et al., 2012). The overactivation of ROS and mitochondrial dysfunction, which result in oxidative stress, play a crucial role in producing VED and ultimately increases cardiovascular risk in OSA (Baltzis et al., 2016; Incalza et al., 2018). According to previous studies, the increased production of ROS correlates with higher interaction with NO and ROS, which form peroxynitrite in one of the earlier periods of endothelial damage (Badran et al., 2014). Mitochondrial dysfunction is associated with the loss of mitochondrial membrane potential and excess production of mitochondrial reactive oxygen species (mitoROS) (Rizwan et al., 2020). Previous studies demonstrated that oxidative stress biomarkers (SOD, GSH-px, and MDA) were increased in rats exposed to CIH (Joseph et al., 2020). Our data confirmed these effects and indicated that As-IV suppressed the activation of inflammation, oxidative stress and mitochondrial dysfunction.

SIRT1 is one of the most extensively studied and vital members of the sirtuin family, and it is an NAD^+^-dependent deacetylase that is highly expressed in endothelial cells (Kida and Goligorsky, 2016). Many studies showed that SIRT1 activators degraded the expression of inflammatory factors and leukocyte adhesion in the aortas of mice and consequently improved VED in cardiovascular diseases (Kida and Goligorsky, 2016; Parsamanesh et al., 2021). SIRT1 also could suppress the development of oxidative stress and mitochondrial dysfunction in endothelial cells (Yuan et al., 2016). A number of recent findings mentioned that SIRT1 mediates eNOS expression via the SIRT1/AMPK pathway (Liu et al., 2016). Our data demonstrated that CIH reduced the level of SIRT1 protein and Thr^172^ AMPK and Ser^1177^ eNOS phosphorylation in the aortas of mice and HCAECs, which were restored by As-IV treatment. To investigate whether SIRT1 was involved in the development of VED, we used isolated aortic rings from control and CIH-exposed mice to measure changes in vascular tension with or without incubation using the SIRT1 activator SRT1720. Treatment with SRT1720 attenuated the impaired EDR to ACh in CIH-exposed aortic rings, and increased the production of NO in HCAECs. SRT1720 treatment also reversed Thr^172^ AMPK and Ser^1177^ eNOS phosphorylation in CIH-treated HCAECs, and the protein expression of total AMPK and eNOS remained unchanged. SRT1720 treatment restrained the increase in inflammatory factors, the activation of oxidative stress and mitochondrial dysfunction in the CIH environment. These results suggested that As-IV suppressed inflammation, oxidative stress and mitochondrial dysfunction via the SIRT1/AMPK/eNOS signaling pathway and eventually ameliorated CIH-induced VED. Calpain-1 is a Ca^2+^-sensitive intracellular cysteine protease that widely exists in endothelial cells (Tang et al., 2015). The overactivation of calpain is involved in VED via the AMPK/eNOS signaling pathway (Miyazaki and Miyazaki, 2018). Convincing evidence revealed that calpains regulate SIRT1 depletion (Biel et al., 2016). Calpain-1 contributes to oxidative stress, mitochondrial dysfunction, inflammation and adhesiveness to leukocytes (Xia et al., 2014; Liu et al., 2020; Dang et al., 2022). Our laboratory and other laboratories reported that calpain-1 aggravated diabetes-induced and arsenic-induced VED (Cai et al., 2019; Nie et al., 2019). However, sufficient evidence to determine whether calpain-1 is participated in CIH-induced VED is lacking. Our study indicated that the expression level of calpain-1 was enhanced in aortas and HCAECs subjected to CIH, and As-IV decreased calpain-1 expression. To test the role of calpain-1 in CIH-induced VED, the thoracic aortic rings in control and CIH-exposed mice with Capn1 knockout were placed in standard organ chambers for isometric tension recording. The results demonstrated that Capn1 knockout improved the EDR impairment induced by CIH. Capn1 knockout reversed NO levels, SIRT1 protein expression and Thr^172^ AMPK and Ser^1177^ eNOS phosphorylation. Notably, Capn1 knockout suppressed the secretion of inflammatory factors, oxidative stress and mitochondrial dysfunction. Taken together, our studies indicated that As-IV ameliorated VED induced by CIH via the calpain-1/SIRT1/AMPK signaling pathway.

## Conclusion

**FIGURE 10:**
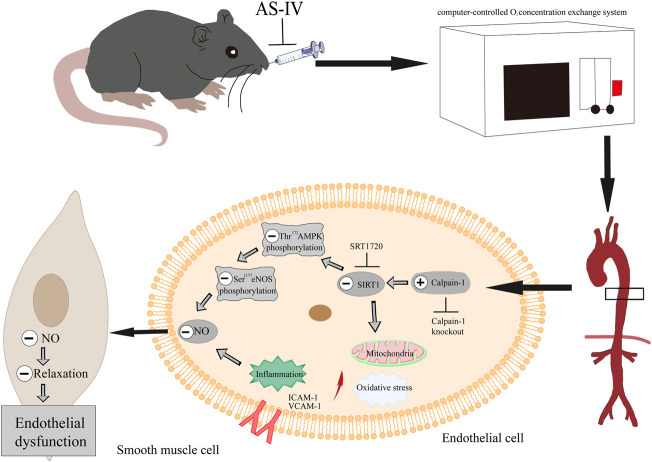
As-IV ameliorates endothelial dysfunction induced by chronic intermittent hypoxia through the calpain-1/SIRT1/AMPK signaling pathway.

In conclusion, these findings confirmed that As-IV ameliorated VED induced by CIH via the calpain-1/SIRT1/AMPK signaling pathway, which suppressed inflammation, oxidative stress and mitochondrial dysfunction, and ultimately protected against the impaired EDR (Figure 10). Our results further provide a novel understanding of AS-IV as a potential treatment for OSA-associated VED.

## Data Availability

The original contributions presented in the study are included in the article/supplementary material, further inquiries can be directed to the corresponding authors.
